# The serotonin transporter gene polymorphism is associated with the susceptibility and the pain severity in Idiopathic Trigeminal Neuralgia patients

**DOI:** 10.1186/1129-2377-15-42

**Published:** 2014-06-20

**Authors:** Wenyao Cui, Xue Yu, Huiqian Zhang

**Affiliations:** 1 Department of Anesthesiology, The First Affiliated Hospital, China Medical University, 110001 NO.155 of Nanjingbei Street, Heping District, Shenyang, Liaoning Province, People’s Republic of China

**Keywords:** Serotonin transporter, Trigeminal Neuralgia, Susceptibility, Chinese

## Abstract

**Background:**

To investigate the possible association between the serotonin transporter gene (5-HTTLPR) and rs 25531 polymorphism and the susceptibility and the pain severity in Trigeminal Neuralgia patients.

**Methods:**

A total of 244 TN patients and 280 age and sex matched healthy volunteer were recruited. 5-HTTLPR and rs 25531 genotyping were performed. All patients received the carbamazepine treatment and the treatment response was evaluated at 6 months.

**Results:**

The genotype distribution of 5-HTTLPR between TN patients and controls were significantly different. The TN Patients had a higher prevalence of short-short genotype than controls. The short-short genotype carriers are also significantly associated with higher pain severity and poorer carbamazepine treatment response compared to the long-long genotype carriers. In contrast, the rs 25531 polymorphism was not associated with the susceptibility to TN, neither with the pain severity and the treat response to carbamazepine.

**Conclusion:**

The 5-HTTLPR polymorphism is associated with the susceptibility to TN and pain severity of TN.

## Background

Trigeminal neuralgia (TN) is the most common type of neuralgia in human adult [1,2]. TN is defined as sudden, usually unilateral, severe, brief, stabbing recurrent episodes of pain within the distribution of one or more branches of the trigeminal nerve [3,4]. carbamazepine (CBZ) is currently widely used for the treatment of TN, although treatment response varies among individual patient [5-8].

Serotonin (also known as 5-hydroxytryptamine, 5-HT) is an important neuromodulator associated with a wide range of physiological effects in the central nervous system. Previous studies have revealed a critical role for 5-HT in numerous physiological processes, including cell division, neuronal migration, differentiation and synaptogenesis. 5-HT has been implicated in a series of pain disorders including migraine, temporomandibular pain and pain conditions mediated by the trigeminal system [9-12]. Serotonin transporter (5-HTT) is a key regulator of 5-HT in neurological system. 5-HTT can inhibit 5-HT release into the synaptic cleft, thereby terminating serotonergic neurotransmission [13].

5-HTT protein is encoded by the *SLC6A4* gene whose transcriptional activity is regulated by a number of variations [13,14]. The serotonin transporter gene (5-HTT)-linked polymorphic region (5-HTTLPR) plays an important role in regulating 5-HTT expression and thereby controlling the concentration of serotonin (5-HT) in brain synapses [15,16]. Recent studies reveal close associations between the genetic variation in the 5-HTTLPR and rs25531 with several pain conditions. For example, 5-HTTLPR gene polymorphism influences the analgesic response to the short acting opioid Remifentanil in human [17]. The 5-HTTLPR polymorphism affects the pain modulation at the supraspinal level [18]. In patients with chronic non-cancer pain, a positive associations between 5-HTTLPR gene polymorphisms and pain perception was reported. Genetic variation in the serotonin transporter gene (5-HTTLPR, rs25531) confers the analgesic response to the short acting opioid Remifentanil in humans [17].

To date, there is no study reporting the association between 5-HTTLPR and the susceptibility to TN and its clinical features, especially the pain severity and treatment response to analgesics. Based on the role played by the 5-HTTLPR gene polymorphisms in other pain conditions [17,18], we postulate that the 5-HTTLPR gene polymorphisms might be also associated with the susceptibility to TN and with its clinical features.

## Methods

### Patients

This study included 244 patients diagnosed with idiopathic TN who were treated at Pain department of the First Affiliated Hospital, China Medical University from August 2008 to August 2012. The TN diagnosis was established according to the International Association for the Study of Pain (IASP) [19]. All patients have paroxysmal attacks of pain, affecting one or more divisions of the trigeminal nerve. Magnetic resonance imaging (MRI) of the whole brain, with particular attention paid to the region of the pons, was done for all subjects to rule out tumor, vascular malformation, multiple sclerosis, or related conditions. Also, complete neurological examinations were given to all patients before treatment to check clinical symptoms. A total of 280 age and sex matched healthy volunteer were recruited from annual checkup visitors as control subjects. All participants were genetically unrelated ethnic Han Chinese people. The exclusion criteria included central nervous system-related disease, pre-existing depression/anxiety, pregnancy, diabetes mellitus, abnormal laboratory baseline values, unstable psychiatric feature (e.g., suicidal attempt), history of alcohol or drug dependence, seizures, neurological illness or concomitant Axis I psychiatric disorder, chronic inflammation disease and cancer. This study received approval from the institutional review boards of the First Affiliated Hospital, China Medical University (2008-A-15B). All patients gave written informed consent to participate in the present study.

### Treatment and pain evaluation

All patients were treated with the traditional approach of CBZ in monotherapy (400–800 mg/day, maximum 1200 mg/day) for 6 months. Patients who discounted the treatment or went to interventional or surgical treatments within 6 months were excluded. The degree of pain was checked using Barrow Neurological Institute [20] scores [20]. Pain intensity scale before and 6 month after treatment was evaluated. The improvement of pain symptom was defined as at least one grade alleviation according BNI scores [21].

### DNA extraction and 5-HTTLPR genotyping

Genomic DNA was extracted from participants' peripheral blood leukocytes using a DNA extraction kit (TianGen, China). The promoter region of the SLC6A4 gene was amplified using a 10 μl of PCR reagent mixture with a forward primer set of 0.25 μM VIC-labeled and 0.25 μM unlabeled, and 0.50 μM reverse primer and 250 μM dNTP on the GeneAmp PCR System 9700 (Applied Biosystems, Foster City, CA, USA). Here, 526 bp and 478 bp fragments were called as a Long and a Short allele at 5-HTTLPR, respectively. For the rs25531 polymorphism, the PCR product was digested with restriction endonuclease MspI (New England Biolabs Inc., Boston, MA, USA). The resulting product was analyzed using an ABI3100 DNA Analyzer and the Peak Scan Software v1.0 (Applied Biosystems). Digested fragments with a size of 164–165 bp were determined as a G allele and indigested as an A allele.

### Statistical analysis

The Fisher's exact Chi-square test was first used to compare the frequency distribution of age, gender, smoking status between TN patients and controls, if appropriate. A chi square test was performed to assess Hardy–Weinberg equilibrium in the TN patients and controls based on allelic and genotypic frequencies. We performed multivariate logistic regression analysis to estimate the effect of 5-HTTLPR or rs25531 polymorphisms on risk factor for TN in the presence of other known prognostic factors, including age, sex and smoke. Analyses were performed using the software SPSS 16.0 (SPSS Inc., Chicago, IL, USA). All P values were two-sided, and a P value < 0.05 was considered significant.

## Results

The characteristics of patients and controls are listed in Table 1. The TN groups had a higher percentage of current smokers (P < 0.001), while the mean age, sex distribution were similar (both P > 0.05).

**Table 1:** The characteristics of patients and controls

	***TN (n = 244)***	***Control (n = 280)***	***P***
*Characteristics*			
***Mean age (yrs)***	*55.06 (38.7-67.8)*	*54.93 (38.5-69.1)*	*0.876*
*Sex*			
*Male*	*100*	*112*	*0.432*
*Female*	*144*	*168*	
***Smoking status***			
*Never*	*70*	*115*	*<0.001*
*Ever*	*94*	*87*	
*Current*	*80*	*78*	
***Symptom Duration (months)***	*40.3 (2.6-56.7)*		
*Pain location*			
*V1*	*10*		
*V2*	*127*		
*V3*	*107*		
***Pain severity***			
*<III*	*81*		
*>TTT*	*163*		

The genotype distribution of 5-HTTLPR between TN patients controls are presented in Table 2. The 5-HTTLPR genotype frequencies in the control group were in Hardy–Weinberg equilibrium (P = 0.486), although those of TN group were different from those expected under Hardy–Weinberg equilibrium (P = 0.005). The rs25531 polymoprhimic distributions in TN and control groups were in Hardy–Weinberg equilibrium (both P > 0.05). TN Patients had a higher prevalence of 5-HTTLPR short-short genotype than controls (35.66% vs. 17.14%, P < 0.001). For allele analyses, TN patients had higher 5-HTTLPR short allele frequency than controls (55.94% vs. 40.18%, P = 0.045). Multivariate regression analyses showed that the short-short genotype carriers had a significantly higher risk for TN development after adjustments with age, sex, and smoke (adjusted OR = 3.219, P < 0.001). With long allele as reference, the OR for 5-HTTLPR short allele carriers was 1.891 (P = 0.045). However, the polymorphism at rs25531 was not significant different between the TN patients and controls (both P > 0.05, Table 2).

**Table 2:** The genotype distribution of HTTLPR between TN patients controls

***Genotype***		***TN***		***Control***		***Adjusted OR***	***95% CI***		***Adjusted P***
*5-HTTLPR*	*Long-long*	*58*	*23.77%*	*103*	*36.79%*	*1.000*			
	*Long-short*	*99*	*40.57%*	*129*	*46.07%*	*1.363*	*0.900*	*2.064*	*0.143*
	*Short-short*	*87*	*35.66%*	*48*	*17.14%*	*3.219*	*1.997*	*5.187*	*0.000*
	*Long*	*215*	*44.06%*	*335*	*59.82%*	*1.000*			
	*short*	*273*	*55.94%*	*225*	*40.18%*	*1.891*	*1.478*	*2.418*	*0.045*
rs25531	*AA*	68	27.87%	75	26.79%	1.000			
	*AG*	114	46.72%	135	48.21%	0.931	0.617	1.406	0.735
	*GG*	62	25.41%	70	25.00%	0.977	0.608	1.569	0.923
	*A*	250	51.23%	285	50.89%	1.000			
	*G*	238	48.77%	275	49.11%	0.987	0.774	1.258	0.913

Before starting treatment, the TN patients were then divided into two groups according to the pain severity: those with severer pain severity (BMI grade: IV-V) and those with less pain (BMI grade: I-III). TN Patients with higher BNI scores had a higher prevalence of 5-HTTLPR short-short genotype and short allele than those with lower BNI scores (31% vs.15%, P < 0.001, Table 3). Multivariate regression analyses showed that the 5-HTTLPR short-short genotype carriage had a dramatically increased higher chance for higher BNI score after adjustments with age, sex and smoking status (adjusted OR = 5.1, P < 0.001). With long allele as reference, the short allele carriage represents significant higher risk for severer pain in TN patients (adjusted OR = 2.146, P = 0.034). In contrast, the polymorphism at rs25531 did not correlate to the BNI score in this study (both P > 0.05, Table 3).

**Table 3:** The genotype distribution of 5-HTTLPR gene polymorphism between TN patients stratified by the pain severity

***Genotype***		***TN with BNI grade (IV-V)***		***TN with BNI grade scores (I-III)***		***Adjusted OR***	***95% CI***		***Adjusted P***
*5-HTTLPR*	*Long-long*	*40*	*24.54%*	*34*	*41.98%*	*1.000*			
	*Long-short*	*75*	*46.01%*	*39*	*48.15%*	*1.635*	*0.898*	*2.975*	*0.107*
	*Short-short*	*48*	*29.45%*	*8*	*9.88%*	*5.100*	*2.122*	*12.260*	*0.000*
	*Long*	*155*	*47.55%*	*107*	*66.05%*	*1.000*			
	*short*	*171*	*52.45%*	*55*	*33.95%*	*2.146*	*1.451*	*3.174*	*0.034*
rs25531	*AA*	35	21.47%	17	20.99%	1.000			
	*AG*	96	58.90%	41	50.62%	1.137	0.573	2.256	0.713
	*GG*	32	19.63%	23	28.40%	0.676	0.307	1.488	0.329
	*A*	166	50.92%	75	46.30%	1.000			
	*G*	160	49.08%	87	53.70%	0.831	0.570	1.212	0.336

All patients were administrated carbamazepine treatment and the pain severity was evaluated 6 months later. A total of 17 patients discontinued the therapy due to the side effect of carbamazepine and another 10 patients underwent interventional or surgical treatments. Thus, a total of 217 patients were evaluated at the end of 6 month, of which 141 patients were assigned to improvement group while the 76 were assigned to non-improvement group based on their BNI scores. The doses of carbamazepine are shown in Table 4. There was no significant dose differences were noted. The genotypes of 5-HTTLPR and rs25531 gene polymorphism are shown in Table 5. TN patients without improvement tent to have significantly higher percentage of short-short genotype and short allele frequencies compare to those with improvement. Multivariate regression analyses revealed that the short-short genotype and short allele are associated with higher risk for treatment failure (adjusted OR = 2.993, P = 0.009 and adjusted OR = 1.672, P = 0.011). In contrast, the rs25531 polymorphism did not affect the treatment response to carbamazepine in this study.

**Table 4:** Summary of dose of carbamazepine and therapy adherence of TN patients

**Dose**	***Treatment without improvement***	***Treatment with improvement***	**P**
400 mg/day	*34*	55	0.620
800 mg/day	*33*	71	
>800 mg/day	*9*	15	
Discontinuation due to side effect	11	6	0.568
Discontinuation due to interventional or surgical treatment	6	4	0.448

**Table 5:** The genotype distribution of 5-HTTLPR and rs25531 gene polymorphism between TN patients stratified by treatment response

***Genotype***		***Treatment without improvement***		***Treatment with improvement***		***Adjusted OR***	***95% CI***		***Adjusted P***
*5-HTTLPR*	*Long-long*	15	19.74%	46	32.62%	1.000			
	*Long-short*	39	51.32%	72	51.06%	1.661	0.824	3.349	0.154
	*Short-short*	22	28.95%	23	16.31%	2.933	1.285	6.696	0.009
	*Long*	69	45.39%	164	58.16%	1.000			
	*short*	83	54.61%	118	41.84%	1.672	1.124	2.488	0.011
rs25531	*AA*	13	17.11%	31	21.99%	1.000			
	*AG*	45	59.21%	76	53.90%	1.412	0.670	2.975	0.363
	*GG*	18	23.68%	34	24.11%	1.262	0.532	2.994	0.597
	*A*	71	46.71%	138	48.94%	1.000			
	*G*	81	53.29%	144	51.06%	1.093	0.737	1.623	0.851

## Discussion and conclusions

To the best of our knowledge, this is the first study reported a close association between the 5-HTTLPR gene polymorphism and the susceptibility to TN. In addition, we found that the 5-HTTLPR gene polymorphism is related to the pain severity and the treatment response to carbamazepine monotherapy in Chinese patients.

The association between 5-HT and pain disorder has been reported. Increased 5-HT levels are noted in human masseter muscle associated with pain and allodynia [12] and following temporomandibular joint movement-evoked pain [22]. Injection of exogenous 5-HT evokes hyperalgesia in both humans, which is attenuated by local administration of 5HT receptor antagonists [11,12,23]. Thus, the modulation of 5-HT level may be a therapy target for pain disorders. 5-HTT is one key regulator of 5-HT by removing 5-HT released into the synaptic cleft, thereby terminating serotonergic neurotransmission [14,24].

5-HTT protein is encoded by the *SLC6A4* gene whose transcriptional activity is regulated by a number of variations [14]. The 5-HTTLPR polymorphism has been well characterized to affect 5-HTT expression: The S and L alleles in the 5-HTTLPR result in lower and higher levels of 5-HTT expression, respectively [25]. The S/S carriers exhibit lower expression of 5-HTT coupled with reduced reuptake of 5-HT from the synapse, leading to stronger psycho-pathological reactions to stressful experiences compared to those with the L/L or L/S allele [26]. The short (14-repeat) allele of 5-HTTLPR was shown to have lower transcriptional activity leading to lower neuronal serotonin reuptake [27]. Previous studies have adequately documented the positive correlation between the S/S genetic phenotype and anxiety-related personality and susceptibility to mood disorders [28,29]. A recent study suggests a significant association between the 5-HTTLPR gene polymorphism and the new onset of depression after PM implantation, especially in women or those who were smokers [30].

Previous study showed an close association between the polymorphism of 5-HTTLPR gene and migraine with aura in children [31]. 5-HTT has important effects on thermal pain perception in preclinical studies [32]. Additionally, 5-HTTLPR polymorphisms have been associated with differences in thermal pain perception [33,34]. The short allele of 5-HTTLPR is associated with emotional modulation of pain but not emotional modulation of spinal nociception [18], however, another study argues that no relationship between the polymorphism of 5-HTTLPR and pain perception in fibromyalgia patients and healthy controls [35]. The discrepancy suggests the effect of 5-HTTLPR gene polymorphism might be condition specific. In this study, we found that the gene polymorphism of 5-HTTLPR is closely related to the risk for TN. The short-short genotype had higher chance to develop TN in our studied cohort.

A previous clinical study tested the effect of escitalopram in Denmark patients with peripheral neuropathic pain. There was no significant association between pain relief and the 5-HTTLPR polymorphism, however, the researchers observed a weak tendency that more responders carry the short-short genotype and more non-responders carry the long-long genotype [36]. In contrast to this study, we found in this study that the short-short genotype of 5-HTTLPR was more related to the treatment failure to carbamazepine monotherapy. It should be noticed that in that Demark study, only 48 patients were enrolled. The sample size in our study is much larger, with 244 TN patients. The ethnic difference may also need to be taken in account for this discrepancy.

Previous studies provided evidence that the genetic polymorphism of several metabolic enzymes (e.g., several isoenzymes of CYP450) and drug transporters (MDR1) are associated with the therapeutically response to analgesics in pain condition [37,38]. The evaluation of the gene polymorphism of these genes could give more information about the carbamazepine metabolic profile of each patient and, consequently, the therapeutic efficacy. However, we did not evaluate the association of 5-HTTLPR polymorphism with carbamazepine response stratified by genetic metabolic profiles to estimate the real weight of 5-HTTLPR polymorphism in carbamazepine efficacy. This is a major limitation of this study.

The other limitations of this study should also be addressed. Several limitations should be addressed in this study. Firstly, this is a singe center study with a relatively small size of patient sample. Thus the enrollment bias may occur. Secondly, this study did not go deep to explore the molecular mechanism under which the 5-HTTLPR genotype influences the TN risk and treatment response.

## Competing interests

All authors declare that they no competing interest.

## Authors’ contributions

WC: acquisition of data, analysis and interpretation of data; drafting the manuscript. XY: Polymorphism analyses, collection of data. WC and HZ, Polymorphism analyses, collection of data. WC and XY: conception and design, revising the manuscript. All authors read and approved the final manuscript.

## References

[B1] Bahgat D Ray DK Raslan AM McCartney S Burchiel KJ Trigeminal neuralgia in young adults J Neurosurg 2011 15 1306 1311 21128738 10.3171/2010.10.JNS10781

[B2] Boto GR Trigeminal neuralgia Neurocirugia (Astur) 2010 15 361 372 21042687

[B3] Ritter PM Friedman WA Trigeminal neuralgia. A debilitating pain syndrome Adv Nurse Pract 2009 15 51 52 19999437

[B4] Burchiel KJ Trigeminal neuralgia J Neurosurg 2010 15 756 757 discussion 757 19747049 10.3171/2009.8.JNS091061

[B5] Brisman R Trigeminal neuralgia: diagnosis and treatment World Neurosurg 2011 15 533 534 22251500 10.1016/j.wneu.2011.06.028

[B6] Shakur SF Bhansali A Mian AY Rosseau GL Neurosurgical treatment of trigeminal neuralgia Dis Mon 2011 15 570 582 22036113 10.1016/j.disamonth.2011.09.003

[B7] Shaikh S Yaacob HB Abd Rahman RB Lamotrigine for trigeminal neuralgia: efficacy and safety in comparison with carbamazepine J Chin Med Assoc 2011 15 243 249 21621166 10.1016/j.jcma.2011.04.002

[B8] Siniscalchi A Gallelli L Avenoso T Squillace A De Sarro G Effects of carbamazepine/oxycodone coadministration in the treatment of trigeminal neuralgia Ann Pharmacother 2011 15 e33 21652789 10.1345/aph.1Q013

[B9] D'Andrea G Granella F Alecci M Manzoni GC Serotonin metabolism in cluster headache Cephalalgia 1998 15 94 96 9533605 10.1046/j.1468-2982.1998.1802094.x

[B10] Smith NL Serotonin mechanisms in pain and functional syndromes: management implications in comorbid fibromyalgia, headache, and irritable bowl syndrome - case study and discussion J Pain Palliat Care Pharmacother 2004 15 31 45 15760806 10.1300/j354v18n04_04

[B11] Ernberg M Hedenberg-Magnusson B Kurita H Kopp S Effects of local serotonin administration on pain and microcirculation in the human masseter muscle J Orofac Pain 2006 15 241 248 16913434

[B12] Ernberg M Lundeberg T Kopp S Pain and allodynia/hyperalgesia induced by intramuscular injection of serotonin in patients with fibromyalgia and healthy individuals Pain 2000 15 31 39 10692600 10.1016/s0304-3959(99)00233-x

[B13] Haddley K Bubb VJ Breen G Parades-Esquivel UM Quinn JP Behavioural genetics of the serotonin transporter Curr Top Behav Neurosci 2012 15 503 535 10.1007/7854_2011_186 22261701

[B14] Homberg JR Lesch KP Looking on the bright side of serotonin transporter gene variation Biol Psychiatry 2011 15 513 519 21047622 10.1016/j.biopsych.2010.09.024

[B15] Nobile M Cataldo MG Giorda R Battaglia M Baschirotto C Bellina M Marino C Molteni M A case–control and family-based association study of the 5-HTTLPR in pediatric-onset depressive disorders Biol Psychiatry 2004 15 292 295 15312818 10.1016/j.biopsych.2004.05.018

[B16] Walderhaug E Magnusson A Neumeister A Lappalainen J Lunde H Refsum H Landrø NI Interactive effects of sex and 5-HTTLPR on mood and impulsivity during tryptophan depletion in healthy people Biol Psychiatry 2007 15 593 599 17544379 10.1016/j.biopsych.2007.02.012

[B17] Kosek E Jensen KB Lonsdorf TB Schalling M Ingvar M Genetic variation in the serotonin transporter gene (5-HTTLPR, rs25531) influences the analgesic response to the short acting opioid Remifentanil in humans Mol Pain 2009 15 37 19570226 10.1186/1744-8069-5-37 PMC2717925

[B18] Palit S Sheaff RJ France CR McGlone ST Potter WT Harkness AR McNulty JL Bartley EJ Hoffmann R Monda JK Rhudy JL Serotonin transporter gene (5-HTTLPR) polymorphisms are associated with emotional modulation of pain but not emotional modulation of spinal nociception Biol Psychol 2011 15 360 369 21291949 10.1016/j.biopsycho.2011.01.008

[B19] O'Connor AB Dworkin RH Treatment of neuropathic pain: an overview of recent guidelines Am J Med 2009 15 S22 32 19801049 10.1016/j.amjmed.2009.04.007

[B20] Trog D Yeghiazaryan K Fountoulakis M Friedlein A Moenkemann H Haertel N Schueller H Breipohl W Schild H Leppert D Golubnitschaja O Pro-invasive gene regulating effect of irradiation and combined temozolomide-radiation treatment on surviving human malignant glioma cells Eur J Pharmacol 2006 15 8 15 16806166 10.1016/j.ejphar.2006.05.026

[B21] Kumar S Rastogi S Mahendra P Bansal M Chandra L Pain in trigeminal neuralgia: neurophysiology and measurement: a comprehensive review J Med Life 2013 15 383 388 24701256 PMC3973876

[B22] Kopp S The influence of neuropeptides, serotonin, and interleukin 1beta on temporomandibular joint pain and inflammation J Oral Maxillofac Surg 1998 15 189 191 9461143 10.1016/s0278-2391(98)90867-9

[B23] Ernberg M Voog U Alstergren P Lundeberg T Kopp S Plasma and serum serotonin levels and their relationship to orofacial pain and anxiety in fibromyalgia J Orofac Pain 2000 15 37 46 11203736

[B24] Patkar AA Berrettini WH Hoehe M Hill KP Sterling RC Gottheil E Weinstein SP Serotonin transporter (5-HTT) gene polymorphisms and susceptibility to cocaine dependence among African-American individuals Addict Biol 2001 15 337 345 11900612 10.1080/13556210020077064

[B25] Gibb BE Benas JS Grassia M McGeary J Children's attentional biases and 5-HTTLPR genotype: potential mechanisms linking mother and child depression J Clin Child Adolesc Psychol 2009 15 415 426 19437301 10.1080/15374410902851705 PMC4113083

[B26] Li Q Cellular and molecular alterations in mice with deficient and reduced serotonin transporters Mol Neurobiol 2006 15 51 66 17003521 10.1385/mn:34:1:51

[B27] Heils A Teufel A Petri S Stöber G Riederer P Bengel D Lesch KP Allelic variation of human serotonin transporter gene expression J Neurochem 1996 15 2621 2624 8632190 10.1046/j.1471-4159.1996.66062621.x

[B28] Caspi A Sugden K Moffitt TE Taylor A Craig IW Harrington H McClay J Mill J Martin J Braithwaite A Poulton R Influence of life stress on depression: moderation by a polymorphism in the 5-HTT gene Science 2003 15 386 389 12869766 10.1126/science.1083968

[B29] Laucht M Treutlein J Schmid B Blomeyer D Becker K Buchmann AF Schmidt MH Esser G Jennen-Steinmetz C Rietschel M Zimmermann US Banaschewski T Impact of psychosocial adversity on alcohol intake in young adults: moderation by the LL genotype of the serotonin transporter polymorphism Biol Psychiatry 2009 15 102 109 19358979 10.1016/j.biopsych.2009.02.010

[B30] Xu H Zhang Q Hou X Wang Q Xu Y Li L Wang N The effect of the polymorphisms of 5-HTTLPR on new onset of depression in patients who underwent pacemaker implantation Psychiatr Genet 2014 15 2 70 4 24077031 10.1097/YPG.0000000000000012

[B31] Szilagyi A Boor K Orosz I Szantai E Szekely A Kalasz H Sasvari-Szekely M Farkas V Contribution of serotonin transporter gene polymorphisms to pediatric migraine Headache 2006 15 478 485 16618266 10.1111/j.1526-4610.2006.00379.x

[B32] Palm F Mössner R Chen Y He L Gerlach M Bischofs S Riederer P Lesch KP Sommer C Reduced thermal hyperalgesia and enhanced peripheral nerve injury after hind paw inflammation in mice lacking the serotonin-transporter Eur J Pain 2008 15 790 797 18187350 10.1016/j.ejpain.2007.11.009

[B33] Lindstedt F Lonsdorf TB Schalling M Kosek E Ingvar M Perception of thermal pain and the thermal grill illusion is associated with polymorphisms in the serotonin transporter gene PLoS One 2011 15 e17752 21423614 10.1371/journal.pone.0017752 PMC3057988

[B34] Lindstedt F Berrebi J Greayer E Lonsdorf TB Schalling M Ingvar M Kosek E Conditioned pain modulation is associated with common polymorphisms in the serotonin transporter gene PLoS One 2011 15 e18252 21464942 10.1371/journal.pone.0018252 PMC3065474

[B35] Potvin S Larouche A Normand E de Souza JB Gaumond I Marchand S Grignon S No relationship between the ins del polymorphism of the serotonin transporter promoter and pain perception in fibromyalgia patients and healthy controls Eur J Pain 2010 15 742 746 20080425 10.1016/j.ejpain.2009.12.004

[B36] Brasch-Andersen C Møller MU Christiansen L Thinggaard M Otto M Brøsen K Sindrup SH A candidate gene study of serotonergic pathway genes and pain relief during treatment with escitalopram in patients with neuropathic pain shows significant association to serotonin receptor2C (HTR2C) Eur J Clin Pharmacol 2011 15 1131 1137 21614492 10.1007/s00228-011-1056-x

[B37] Lotsch J Skarke C Liefhold J Geisslinger G Genetic predictors of the clinical response to opioid analgesics: clinical utility and future perspectives Clin Pharmacokinet 2004 15 983 1013 15530129 10.2165/00003088-200443140-00003

[B38] Samer CF Piguet V Dayer P Desmeules JA Genetic polymorphism and drug interactions: their importance in the treatment of pain Can J Anaesth 2005 15 806 821 16189332 10.1007/BF03021775

